# 8-Hydroxy-2’-deoxyguanosine as a biomarker of oxidative stress in acute exacerbation of chronic obstructive pulmonary disease

**DOI:** 10.3906/sag-1807-106

**Published:** 2019-02-11

**Authors:** Xing LIU, Kaili DENG, Sixia CHEN, Yunshi ZHANG, Jing YAO, Xiaoqin WENG, Yang ZHANG, Tianming GAO, Ganzhu FENG

**Affiliations:** 1 Department of Respiratory Medicine, Second Affiliated Hospital of Nanjing Medical University, Nanjing P.R. China; 2 Department of Tuberculosis, Xuzhou Infectious Disease Hospital, Xuzhou P.R. China

## 1. Introduction

Chronic obstructive pulmonary disease (COPD) is characterized by progressive airflow limitation and this pathologic change is poorly reversible. The airflow limitation is associated with structural remodeling of small airways, which can be precipitated by pollutants such as toxic gases and harmful particulates (1,2). The progression of COPD is variable. Some patients experience relatively stable courses while others suffer frequent acute exacerbations (3,4). The frequency of acute exacerbation accelerates the decline of lung function, which not only impairs the quality of life but also increases health care utilization (1,5,6). Therefore, prevention, diagnosis, and treatment of acute exacerbation are crucial for COPD patients.

Cytokines and inflammatory factors are thought to play an important role in the progression of COPD (7,8) and act as marks of pathologic changes in the lungs during disease progression. Acute exacerbation of COPD (AECOPD) is associated with increased inflammation, involving the accumulation of different inflammatory cells and mediators in both lung tissue and circulation (9,10). Besides, oxidative stress has also been recognized to play an important role in the pathogenesis of COPD (11,12), and the extent of oxidative stress can be estimated by several biomarkers such as glutathione (GSH), total superoxide dismutase (SOD), and malondialdehyde (MDA) (13,14). Therefore, detection of these biomarkers may be helpful in evaluating the severity of COPD.

8-Hydroxy-2’-deoxyguanosine (8-OHdG), continuously produced in living cells, is an end-product of oxidative DNA damage. It is one of the most frequently used biomarkers for evaluating oxidative stress. Previous studies have observed elevated 8-OHdG in diabetes, cancer, cardiovascular diseases, and stable COPD (15–17). However, the level of 8-OHdG in AECOPD is still unclear. The purpose of our study was to explore plasma 8-OHdG level in patients with AECOPD and its clinical value.

## 2. Materials and methods

### 2.1. General data

A total of 134 patients with COPD admitted to the Second Affiliated Hospital of Nanjing Medical University between September 2015 to August 2016 were included in this study. At the same time, 20 healthy volunteers with normal pulmonary function were enrolled in the study. Ethical approval was given by the Ethics Committee of the Second Affiliated Hospital of Nanjing Medical University and written informed consent was obtained from all subjects.

The diagnosis and severity assessment of COPD were based on the Global Initiatives for Chronic Obstructive Lung Disease (GOLD) guidelines, and acute exacerbation was defined as an acute worsening condition from stable state, which presented with aggravated dyspnea, increased sputum volume, and/or changed sputum color, or any combination of these symptoms and requirement to alter the treatment plan (5). Among the COPD patients included, 24 patients were in a clinically stable phase and 110 patients underwent an acute exacerbation. Epidemiological (age, sex, body mass index (BMI)) and clinical data (blood pressure, smoking status, lung function, home oxygen therapy, presence of comorbidities) were collected at the time of recruitment. Pulmonary function tests were conducted for a formal diagnosis of COPD. The severity of the disease was categorized as GOLD stage 1, mild (FEV1 ≥ 80%); GOLD stage 2, moderate (50% ≤ FEV1 < 80%); GOLD stage 3, severe (30% ≤ FEV1 < 50%); and GOLD stage 4, very severe (FEV1 < 30%) (5). The Modified British Medical Research Council (mMRC) questionnaire (18) on breathlessness and the COPD Assessment Test (CAT) score (19), a comprehensive assessment of symptoms, were recorded for all COPD patients. According to the frequency and severity of acute exacerbations within 1 year, the patients were divided into a low-risk group (exacerbation ≤1 time within 1 year and no hospitalization for exacerbation) and high-risk group (exacerbations ≥2 times or exacerbation ≥1 time and hospitalization for exacerbation within 1 year). Combined COPD assessment (A–D), which highlighted both patient-reported outcomes and the importance of exacerbation prevention in COPD management, was also conducted. Smoking status in our study was based on self-report. Exclusion criteria were as follows: patients with a history of active pulmonary tuberculosis, bronchial asthma, bronchiectasis, or cystic fibrosis, or receiving corticosteroids or antibiotics treatment within 4 weeks. Patients with no adequate plasma sample were also excluded. 

### 2.2. Measurements of circulating 8-OHdG and other biomarkers

Peripheral venous blood samples were obtained at the time of inclusion into the study. Serum procalcitonin (PCT) was quantified by immunofluorescent assay. Serum C-reactive protein (CRP) was estimated by high-sensitivity CRP assay. Serum CD64 index was measured by flow cytometry using a commercial kit. Another 5 mL of peripheral venous blood was collected and centrifuged for 15 min (3000 rpm) at 4 °C within 1 h, and then the supernatant was extracted and cryopreserved at –80 °C for later 8-OHdG analysis. Plasma 8-OHdG concentration was quantified by an enzyme-linked immunosorbent assay (ELISA) kit (Calvin, Suzhou, China). All tests were conducted according to the manufacturer’s instructions.

### 2.3. Statistical analysis

All statistical analyses were performed using SPSS 15.0 (SPSS Inc., Chicago, IL, USA). The continuous variables were expressed as mean ± standard deviation (SD) and discrete variables as counts (percentages). Student’s t-test was used for comparison between two groups and ANOVA was used for comparison among groups. The correlation analyses between 8-OHdG and spirometry results, CRP, PCT, and CD64 were analyzed using Pearson’s test. P < 0.05 was considered statistically significant.

## 3. Results

### 3.1. Demographic information and clinical characteristics 

The demographic information and clinical characteristics of the study population are summarized in the Table. There were no significant differences in age, sex, or BMI among the three groups (P > 0.05). Lung function tests were recorded for all included subjects. In both COPD groups, FEV1, FEV1% predicted, and FEV1/FVC were significantly lower in comparison with those in healthy group. In addition, blood pressure, smoking status and associated comorbidities of all subjects are shown in the Table.

**Table 1:** The demographic and clinical characteristics of study subjects.

Characteristics	Healthy subjects(n = 20)	Stable COPD(n = 24)	AECOPD(n = 110)
Age (years)	64.34 ± 8.37	65.43 ± 6.90	68.21 ± 7.82
Sex (male/female)	13/7	13/11	62/48
BMI (kg/m2)	27.68 ± 5.53	24.22 ± 6.12	25.21 ± 5.31
Systolic blood pressure (mmHg)	129.4 ± 5.69	132.12 ± 8.96	152.05 ± 10.02*
Diastolic blood pressure (mmHg)	63.70 ± 2.69	60.65 ± 1.68	62.91 ± 4.18
Smoking status			
Current smokers, n (%)	9 (45.00)	10 (41.67)	23 (20.91)
Ex-smokers, n (%)	4 (20.00)	3 (12.50)	41 (37.27)
Nonsmokers, n (%)	7 (35.00)	11 (45.83)	46 (41.82)
Smoking history (years)	42 ± 10.61	43 ± 18.06	45 ± 15.32
GLOD stage (1/2/3/4), n	-	2/8/11/3	12/33/50/15
FEV1 (L)	3.41 ± 0.30	2.15 ± 0.22*	1.86 ± 0.72*#
FEV1 % predicted	85 ± 8.85	59.81 ± 10.13*	48.63 ± 10.35*
FVC (L)	3.84 ± 0.57	3.63 ± 0.81	3.37 ± 0.69*
FEV1/FVC	0.88 ± 0.14	0.61 ± 0.14*	0.58 ± 0.22*
mMRC grade (0/1/2/3/4), n	-	4/6/7/5/2	9/21/40/35/5
CAT score	-	18.25 ± 5.61	20.75 ± 4.80
Risk of exacerbations			
Low-risk, n (%)	-	8 (33.33)	34 (28.33)
High-risk, n (%)	-	16 (66.67)	86 (71.67)
Combined COPD assessment (A/B/C/D), n	-	4/5/8/7	15/28/36/31
Home oxygen therapy, n (%)	-	1 (4.17)	6 (5.45)
Comorbidities, n (%)			
Cardiovascular diseases	-	16 (66.67)	86 (78.18)
Tumor	-	1 (4.17)	8 (7.27)
Pneumonia	-	1 (4.17)	42 (38.18)
Diabetes	-	6 (25.00)	25 (22.73)
Cerebrovascular disease	-	5 (20.83)	50 (45.45)
Liver disease	-	1 (4.17)	11 (10.00)
Renal disease	-	4 (16.67)	23 (20.91)

Data are presented as mean ± SD, unless otherwise indicated. * P < 0.05 compared with healthy group; # P < 0.05, compared with stable COPD group.

### 3.2. Plasma 8-OHdG levels were upregulated in AECOPD patients

**Figure 1:**
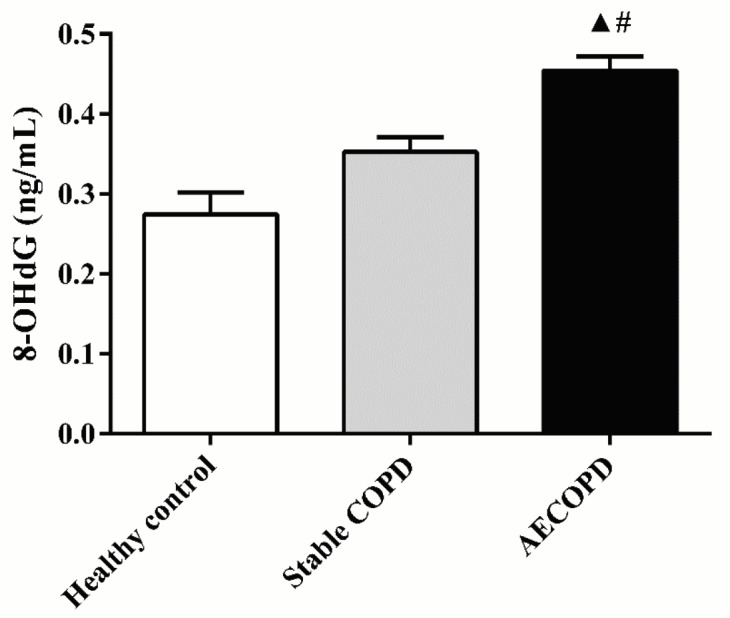
Levels of plasma 8-OHdG from healthy volunteers, stable COPD patients, and AECOPD patients. ▲ P < 0.05 compared with healthy group; # P < 0.05 compared with stable COPD group.

Plasma 8-OHdG concentrations in healthy, stable COPD, and AECOPD subjects are shown in Figure 1. We found that 8-OHdG levels in AECOPD patients were increased significantly in comparison with those in both stable COPD patients and healthy subjects (0.45 ± 0.19 vs. 0.35 ± 0.10 ng/mL, P < 0.05; 0.45 ± 0.19 vs. 0.27 ± 0.18 ng/mL, P < 0.05, respectively). However, no statistical difference of 8-OHdG was observed between stable COPD patients and healthy subjects (P > 0.05).

### 3.3. 8-OHdG levels were further elevated in AECOPD patients with smoking history

**Figure 2:**
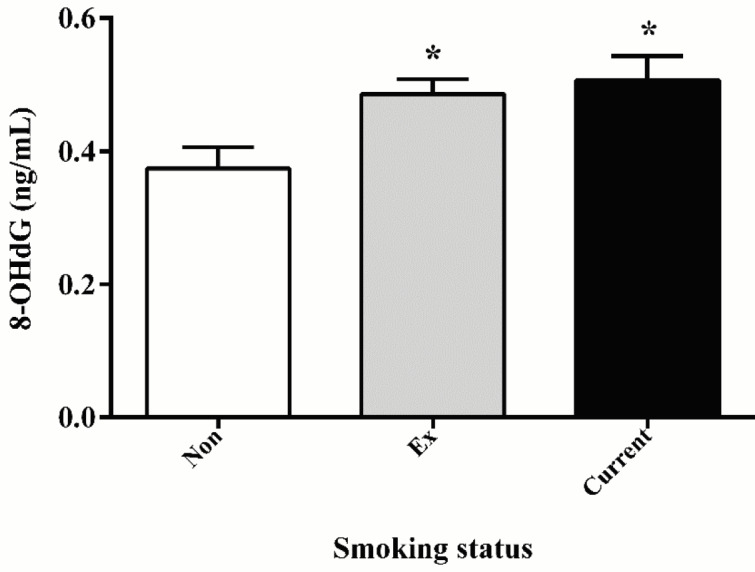
Levels of plasma 8-OHdG of AECOPD patients of different smoking statuses. * P < 0.05 compared with nonsmokers; “Non”, “Ex”, and “Current” represent nonsmokers, ex-smokers, and current smokers, respectively.

As shown in Figure 2, plasma 8-OHdG levels in patients with AECOPD of different smoking statuses were investigated. Only low levels of 8-OHdG were detected in nonsmokers (0.37 ± 0.20 ng/mL). In contrast, high levels of 8-OHdG were detected in both ex- and current smokers (0.49 ± 0.17 ng/mL and 0.51 ± 0.18 ng/mL, respectively). However, there was no significant difference between ex- and current smokers in plasma 8-OHdG level (P = 0.64). 

### 3.4. 8-OHdG level was associated with lung function grade in AECOPD patients

**Figure 3:**
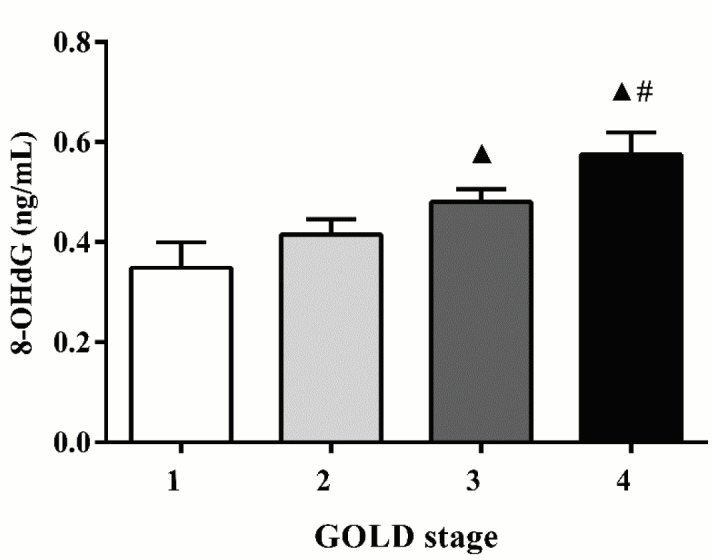
Levels of plasma 8-OHdG in patients with AECOPD of different GOLD stages. ^▲^ P < 0.05 compared with GOLD stage 1; # P < 0.05 compared with GOLD stage 2.

The relationship between the level of 8-OHdG and the severity of GOLD stage is shown in Figure 3. We found that plasma 8-OHdG levels were significantly higher in AECOPD patients with GOLD stage 3 and 4 than those with GOLD stage 1 (0.48 ± 0.18 vs. 0.35 ± 0.19 ng/mL, P < 0.05; 0.58 ± 0.19 vs. 0.35 ± 0.19 ng/mL, P < 0.05, respectively) and 8-OHdG levels were higher in GOLD stage 4 patients compared with those in GOLD stage 2 (0.58 ± 0.19 vs. 0.41 ± 0.18 ng/mL, P < 0.05). 

### 3.5. 8-OHdG level was associated with the severity of symptoms in AECOPD patients

**Figure 4:**
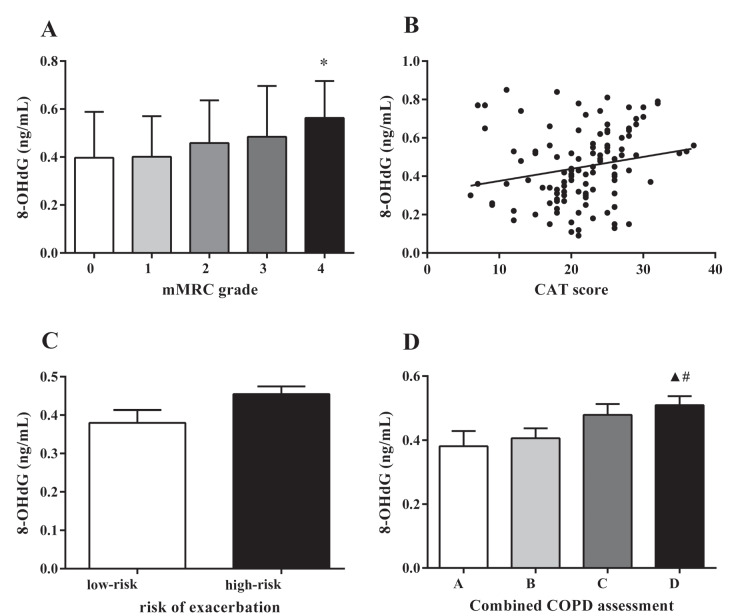
Correlations between 8-OHdG levels and mMRC grade, CAT score, risk of exacerbations, and combined COPD assessment in AECOPD patients. * P < 0.05 compared with mMRC grade 1; ▲ P < 0.05 compared with group A; # P < 0.05 compared with group B.

The relationships between 8-OHdG and mMRC grade, CAT score, and risk of exacerbation were explored. The results showed that greater mMRC grade had a higher trend with 8-OHdG level and the 8-OHdG level in the mMRC grade 4 group was significantly higher than that in the mMRC grade 1 group (P = 0.04) (Figure 4A). Positive correlation was observed between 8-OHdG level and CAT score (r = 0.206, P = 0.027) (Figure 4B). Higher level of 8-OHdG was observed in the high-risk group (high-risk group vs. low-risk group, 0.45 ± 0.02 vs. 0.38 ± 0.03 ng/mL), but no statistically significance was found (P = 0.701) (Figure 4C). The exploration of 8-OHdG level and combined COPD assessment showed that 8-OHdG was markedly elevated in group D compared with those in groups A and B (0.38 ± 0.05 ng/mL vs. 0.51 ± 0.03, 0.41 ± 0.03 vs. 0.51 ± 0.03 ng/mL, P < 0.05) (Figure 4D).

### 3.6. 8-OHdG in AECOPD patients was negatively correlated with spirometry results but positively correlated with inflammatory markers 

**Figure 5:**
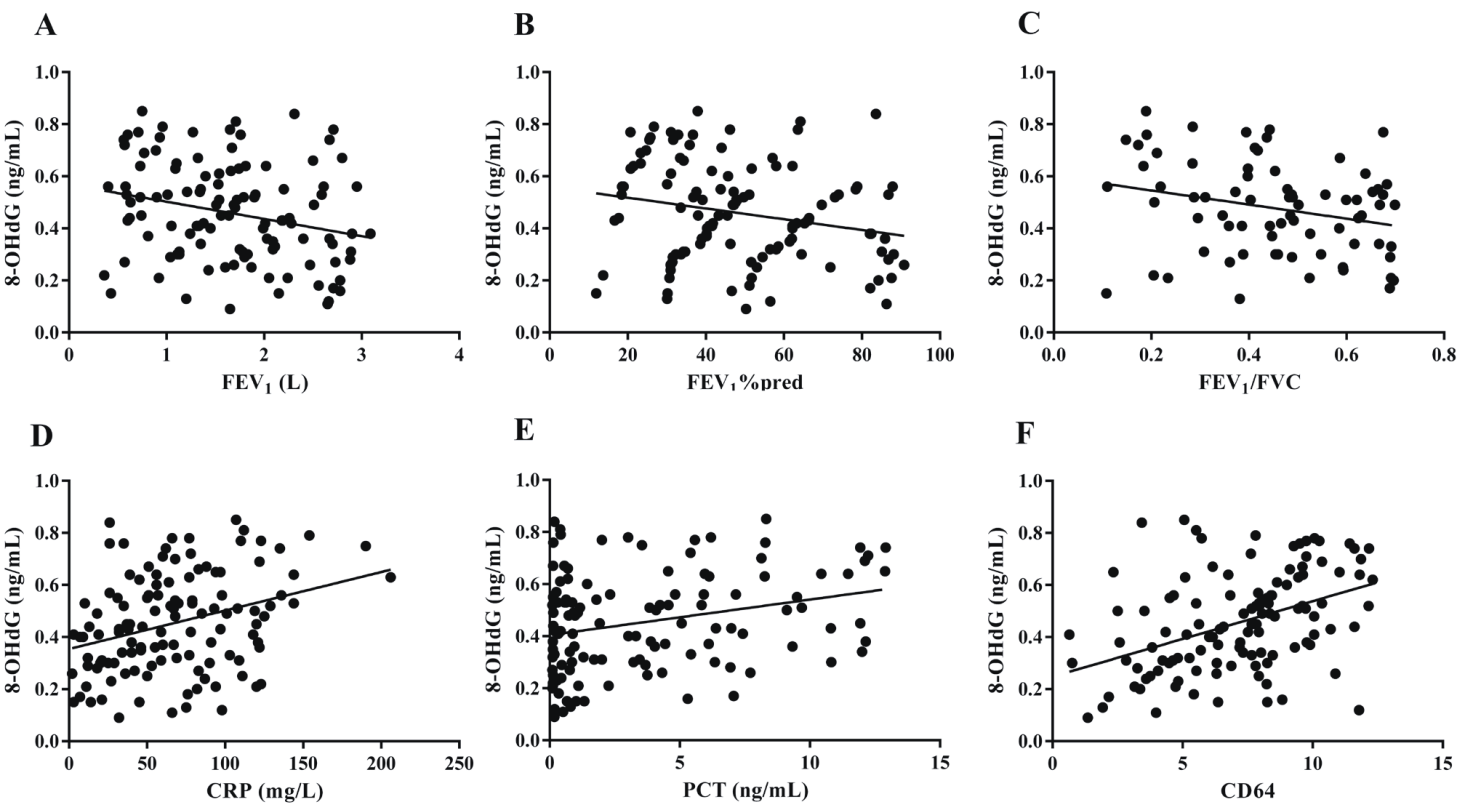
Correlations between 8-OHdG levels and spirometry results and inflammatory biomarkers in AECOPD patients.

The correlations between 8-OHdG and spirometry results in AECOPD patients were explored. In the univariate analysis, negative correlations between 8-OHdG and FEV1 (r = –0.25, P = 0.007), FEV1% predicted (r = –0.22, P = 0.018), and FEV1/FVC (r = –0.242, P = 0.009) were observed (Figures 5A–5C). The correlations between 8-OHdG and inflammatory biomarkers were also explored. As shown in Figures 5D–5F, there were positive correlations between 8-OHdG and CRP (r = 0.325, P = 0.000), PCT (r = 0.276, P = 0.002), and CD64 (r = 0.423, P = 0.000).

## 4. Discussion

COPD is an aberrant inflammatory disease in airways, pulmonary parenchyma, and vessels that results from exposure to cigarette smoke, inhaled toxins, and gene–environment interactions (5,8,9,18). Like other chronic diseases, COPD presents with not only organ-specific characteristics but also systemic manifestations (20,21). Acute exacerbations are significant events in the natural history of COPD, which contribute to tremendous mortality all over the world. Oxidant/antioxidant imbalance by lung cytotoxic mechanisms has been demonstrated to play an important role in the pathogenesis of COPD (22–24). Oxidative balance is necessary for normal physiological functions, but inhaled pollutants and other chemical factors can disturb this balance. Under normal state, endogenous antioxidants, such as superoxide dismutase, glutathione peroxidase, and catalase, protect living organisms from oxidative damage (13). However, excessive oxidative stress leads to mitochondrial DNA damage as well as cellular injury (25).

8-OHdG, an oxidized form of guanine that is produced from deoxyguanosine in DNA by reactive oxygen species (ROS), was first reported in 1984 by Kasai et al. (15,26). The level of 8-OHdG can be used for estimating oxidative DNA damage. Previous studies have demonstrated that 8-OHdG level increases in many diseases, such as diabetes, cancer, and cardiovascular diseases (15–17). Our study showed that plasma 8-OHdG levels were elevated in AECOPD patients compared with those in stable COPD and healthy subjects, but no statistical difference was found between stable COPD patients and heathy subjects. This finding suggests that oxidant stress increases during AECOPD and application of 8-OHdG can be an appropriate approach to diagnose AECOPD, monitor disease progression, and guide treatment. 

Increasing experimental evidence has proved that smoking damages the lung tissue by the means of direct toxicity to bronchial epithelial cells, oxidative damage, and chronic inflammation (27,28). Cigarette smoke contains high concentrations of free radicals and oxidants, which are the major exogenous predisposing factors causing ROS production as well as inflammation (29,30). According to Asami et al. (31), 8-OHdG is elevated in the lungs and peripheral leukocytes of smokers. In our study, the plasma concentration of 8-OHdG was detected in different smoking statuses during AECOPD. We found that 8-OHdG was increased in smoking patients in comparison with nonsmoking patients, indicating that smoking exacerbated the oxidative damage in AECOPD. Therefore, it is important to stop smoking to reduce damage in lung tissue. However, no statistical difference in 8-OHdG concentration was observed between ex- and current smokers in AECOPD patients, suggesting that oxidative DNA damage continued even after smoking cessation (32).

Treatments of COPD are centered around patient outcomes including spirometric severity, exacerbation rate, and symptoms according to global strategies for the diagnosis, management, and prevention of COPD (5). However, the relationship between oxidative stress and the disease severity of COPD is still uncertain. Previous research has shown that the imbalance of oxidants/antioxidants not only promotes inflammation process, but also affects airway obstruction adversely and accelerates decline in lung function (33). The present results also provide important evidence for this viewpoint. In our study, 8-OHdG level was positively correlated with the severity of lung function, indicating that excessive oxidative stress accelerated the decline of lung function. This finding suggests that 8-OHdG can be used for evaluating the spirometric severity of AECOPD. Further study revealed that higher 8-OHdG level was associated with greater mMRC grade, higher CAT score, and higher group level of combined COPD assessment. These findings suggest that 8-OHdG can be used for evaluating symptomatic severity and exacerbation risk in AECOPD patients.

To our knowledge, about half of the exacerbations of COPD are precipitated by bacterial and viral infections (34). Cytokines and inflammatory factors are available for diagnosis and assessment of inflammatory process in AECOPD (34–37). A number of publications have shown that CRP, PCT, and CD64 are sensitive biomarkers for bacterial infections in AECOPD (37). During inflammatory stimulation, their expressions are rapidly upregulated. Brusselle et al. (38) reported that enhanced oxidative stress causes increased systemic inflammation in the airways during AECOPD. In our study, significant positive correlations between plasma 8-OHdG level and inflammatory biomarkers were observed, which confirmed the findings that oxidative stress and systemic inflammation were strongly interrelated processes during AECOPD (38–40). These observations also indicate that combined measurements of different biomarkers can be useful in understanding and management of AECOPD patients.

Our study had some limitations. First, the numbers of stable COPD patients and healthy volunteers were insufficient, and larger number of subjects are needed to draw robust conclusions. Second, we did not conduct any study involving molecular mechanisms. Lastly, we did not collet a second plasma sample from AECOPD patients during hospitalization or discharge; thus, the outcomes of patients with AECOPD could not be assessed by 8-OHdG. In conclusion, the present study investigated plasma 8-OHdG levels in AECOPD for the first time. The 8-OHdG level was found to increase in patients with AECOPD and was higher in smokers. It was not only associated with spirometric severity, symptomatic severity, and exacerbation risk, but was also positively correlated with C-reactive protein, procalcitonin, and neutrophil CD64. These findings suggest 8-OHdG as a promising biomarker to guide the choice of optimal therapeutic directions. 

## Acknowledgments

This study was supported by the Natural Science Foundation of China (No. 81670013). We are grateful to the clinical staff in the Department of Respiratory Medicine of the Second Affiliated Hospital of Nanjing Medical University, the healthy volunteers, and the patients included.
